# Supplementary material for: State Optimism as a State-Dependent Prior About Rewarding Outcomes
Source: Comput Brain Behav. 2025 Aug 11;9(1):133–53. doi: 10.1007/s42113-025-00256-6 (PMC13293035; doi:10.1007/s42113-025-00256-6)
Supplement: Supplementary file 1 — Supplementary file1 (DOCX 1780 KB) [file 42113_2025_256_MOESM1_ESM.docx]

**Supplementary information:**

**State optimism as a state-dependent prior about rewarding outcome**

**Authors & Affiliations**

Kanji Shimomura^1,2^* & Kenji Morita^1,3^

1 Graduate School of Education, The University of Tokyo, Tokyo, Japan

2 Japan Society for the Promotion of Science, Tokyo, Japan

3 International Research Center for Neurointelligence (WPI-IRCN), The University of Tokyo, Japan

*Corresponding author

Kanji Shimomura (skcpccp@gmail.com, ORCID: 0000-0003-4370-3710)

**Participants’ motivation for and focus on imagery exercises**

To assess engagement in imagery exercises and possible between-group differences in it, we calculated within-participants mean levels of motivation and focus across two weeks of the intervention, and compared them between the BPS and control groups. As stated in the main text, participants in both groups generally showed high motivation and moderate focus, with no significant between-group differences in both indices (Fig. S1).


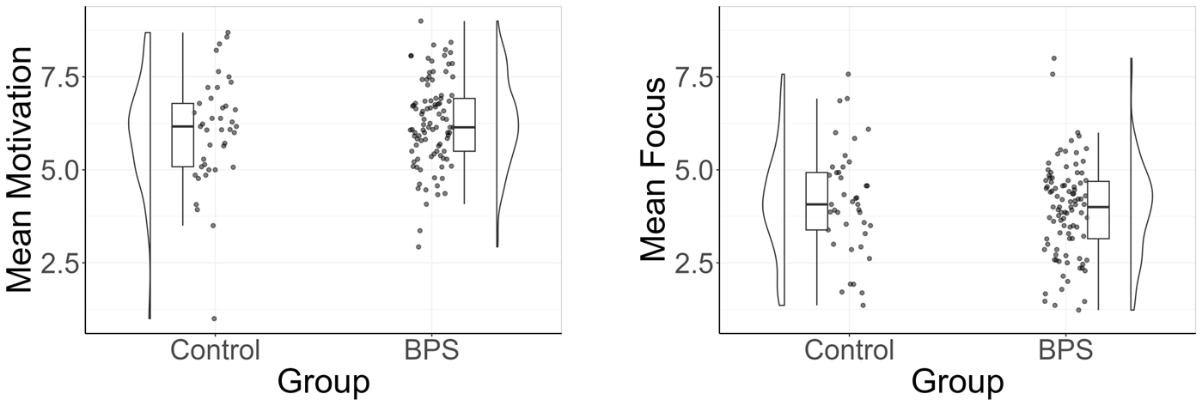


**Fig. S1** Motivation (left) and focus (right) regarding imagery exercise across two weeks.

**Manipulation of presented base rates in the news task**

As stated in the main text, we systematically manipulated the presented base rates to control the size of estimation errors in GOOD and BAD trials. We adopted almost the same way as used in previous study (Kuzmanovic et al., 2017). In short, we prepared a list of estimation errors for 20 trials (10 GOOD and 10 BAD trials) in advance. For GOOD trials, we subtracted the corresponding value from the estimated base rate of participants, while for BAD trials, we added it to the estimated base rate. When the resulting base rate was inappropriate to present (i.e., less than 1% or higher than 90%), the experimental program searched for other values from the list until an appropriate base rate was found. If the appropriate value was found in the remaining values in the list, that value was used to calculate the base rate, and the positions of the two values within the list were swapped (i.e., the unused value was used in the later trial). If there were no appropriate values in the list, then 1% was presented in the GOOD trials whereas 90% was presented in the BAD trials. In this case, a realized value of the estimation error was calculated by the difference between the estimated and actual base rate (1% or 90%), and the old, unused, value was replaced with this realized value. To make the estimation errors compatible between GOOD and BAD trials, the realized list of GOOD trials was then used in the subsequent 10 BAD trials, and the realized list of BAD trials was used in the subsequent 10 GOOD trials. For more details, see the supplementary material of Kuzmanovic & Rigoux (2017).

Our version had two modifications from the original one. Firstly, we used smaller values for the list of estimation errors to make the presented actual base rate more credible for participants (i.e., to make it less noticeable that the base rate is being manipulated). Specifically, while the previous study used the values ranging from 5% to 24%, we used the values ranging from 6% to 15%.

Secondly, we limited the answers of participants for likelihood to 1-90%, not 1-99%. This modification was intended to prevent a subtle technical issue. Although the original version allowed participants to answer any integer from 1 to 99, at the same time they set the maximum value of the manipulated base rate to 90% in order not to present unnaturally high base rates. This can lead to a biased number of GOOD trials, although such a situation is rare. Specifically, when a participant’s answer is higher than 90% in a trial whose pre-determined condition is “BAD”, the “actual” base rate should become lower than the estimated base rate (i.e., turning into a “GOOD” trial) because the highest base rate that can be presented is 90%. Such a situation increases the number of GOOD trials relative to that of BAD trials. To avoid this problem, we used the range of 1-90% in our study.

**Specific instructions for the Best Possible Self (BPS) intervention**

We used almost the same instruction used in the previous study (Meevissen et al., 2011). The only difference was that the introductory sentence was slightly revised to avoid hinting at the existence of conditions. Specifically, the opening sentence of the original version was as follows:

*You have been randomly assigned to a condition in which you are going to think of your best possible self (BPS group) / pay more attention to the daily activities of your life (control group).*

We modified this to:

*From now on, you will be asked to imagine your best possible self (BPS group) / your typical behaviors (control group).*

Therefore, the full text of the instructions for each group was as follows:

**Best Possible Self group**:

*From now on, you will be asked to imagine your best possible self. Your best possible self means imagining yourself in a future in which everything has turned out as good as possible. You have worked hard and you have managed to realize all your life goals. You can envision it as satisfying all your life dreams and development of all your best possible potentials. In a moment, you are going to think of the best possible ways in which your life could develop on three domains (Personal, Relational, and Professional), with the goal to direct the decisions you make in the present. You have probably never thought about yourself in this way but research has indicated that this method may have a positive influence on your mood. We would like to encourage you to keep thinking about and imagine yourself in this way during the next two weeks. In order to determine and guide constructing your best possible self, for the next 20 min you are going to think of and write down your goals, skills and desires you would like to achieve in the far future for each of the three domains, and finally merge these into a personal story like a diary. Think during this whole process of realistic skills and manageable goals/wishes that you would like to possess or attain in the future.*

**Personal domain**

*Think of goals you would like to attain on the personal level (e.g. physical and psychological skills and developments).*

**Professional domain**

*Think of goals you would like to attain on the professional/work-related level (e.g. position, accomplishments, level of expertise, but also occupation and skills, etc.).*

**Relational domain**

*Think of goals you would like to attain on the relational level (e.g. relations and contacts with loved ones, friends, colleagues, but also joint activities etc. in your social life).*

*Now we would like to ask you to write down in as much detail as possible your ideal future. You can use the goals you have just constructed as a guide.While describing your thoughts, try to activate your senses, feelings, and perceptions and make a personal story of it.*

**Control group**:

*From now on, you will be asked to imagine your typical behaviors . This means you are going to give more thought to the ordinary daily activities of your life that normally would have gone unnoticed like for instance, specific meetings, lectures, conversations, typical thoughts you have during the day, etc. Use your day schedule of the past 24 h as guidance. This exercise will help you to more easily identify difficult problem areas you may have in your life and take action in order to improve these areas. You have probably never thought about yourself in this way but research has indicated that this method may have a positive influence on your mood. We would like to encourage you to keep thinking about and imagine yourself in this way during the next two weeks. In order to help you determine and guide your focus, work according to the following structure. Think of your day schedule of the past 24 h and go over it calmly. Think of the activities, meetings, etc. and go more deeply into the conversations, discussions, thoughts, or mood you may have had. For the next 20 min we would like to ask you to write down your thoughts. While describing your thoughts, try to activate your senses, feelings, and perceptions and make a personal story of it.*

These instructions were translated into Japanese by a commercial bilingual team who were irrelevant to this study. The translated version was reviewed by the authors of this study. The finalized Japanese version of the instructions is available at <https://osf.io/wvkux/>.

**Results of model comparison in the case where “Leaky models” were added to candidate models**

As stated in main text, we did not include “Leaky model” and its variant in our primary model fitting procedure. However, we examined whether the obtained results are maintained even with the inclusion of the Leaky models. We considered two types of Leaky models as in previous study (Rupprechter et al., 2018): “Basic leaky model” and “Leaky-$\rho$ model”.

In the Basic leaky model, an agent updates the values of stimuli (images) in the following way:

$$\begin{aligned} V_{i}^{t+1}= {AV}_{i}^{t}+ R_{t}\#\left( S1 \right) \end{aligned}$$

where $A$ denotes the forgetting parameter. The initial values were set to 0 for all stimuli. The agent then uses this value to make decisions in a soft-max manner:

$$\begin{aligned} p\left( choose {image}_{i} \right)=\frac{1}{1+\exp\left( -\gamma\times\left( \frac{V_{i}}{4}-{explicit value}_{i} \right) \right)}\#\left( S2 \right) \end{aligned}$$

Note that unlike other models, the learned values were divided by four to scale them to [0, 1]. The only difference between the Basic leaky model and the Leaky-$\rho$ model was that the latter included an additional parameter $\rho$ to represent individual differences in the sensitivity to rewards. Thus, in the Leaky-$\rho$ model, the values were updated in the following way:

$$\begin{aligned} V_{i}^{t+1}= {AV}_{i}^{t}+ {\rho R}_{t}\#\left( S3 \right) \end{aligned}$$

We conducted the same model-fitting procedure as stated in the main text, with these two additional candidate models. We confirmed that the same model, i.e., the basic Bayesian model, was selected as the best model in both groups at both time points (all PXPs > 0.99). Therefore, the conclusion is robust regardless of the inclusion of Leaky models.

**Illustration of beta distributions**

To provide an intuitive understanding of the properties of the beta distribution, we illustrated the beta distribution with various parameter values (Fig. S2). The figures are arranged from left to right with means of 1/3, 1/2, and 2/3, respectively. Additionally, the figures in the lower rows are arranged to have $\alpha$ and $\beta$ values that are ten times those of the row above, with the same mean. As the relative value of $\alpha$ compared to $\beta$ increases, the mean of the distribution gets higher. Also, it can be seen that the figures in the lower rows have smaller variance compared to the corresponding figures in the upper rows.


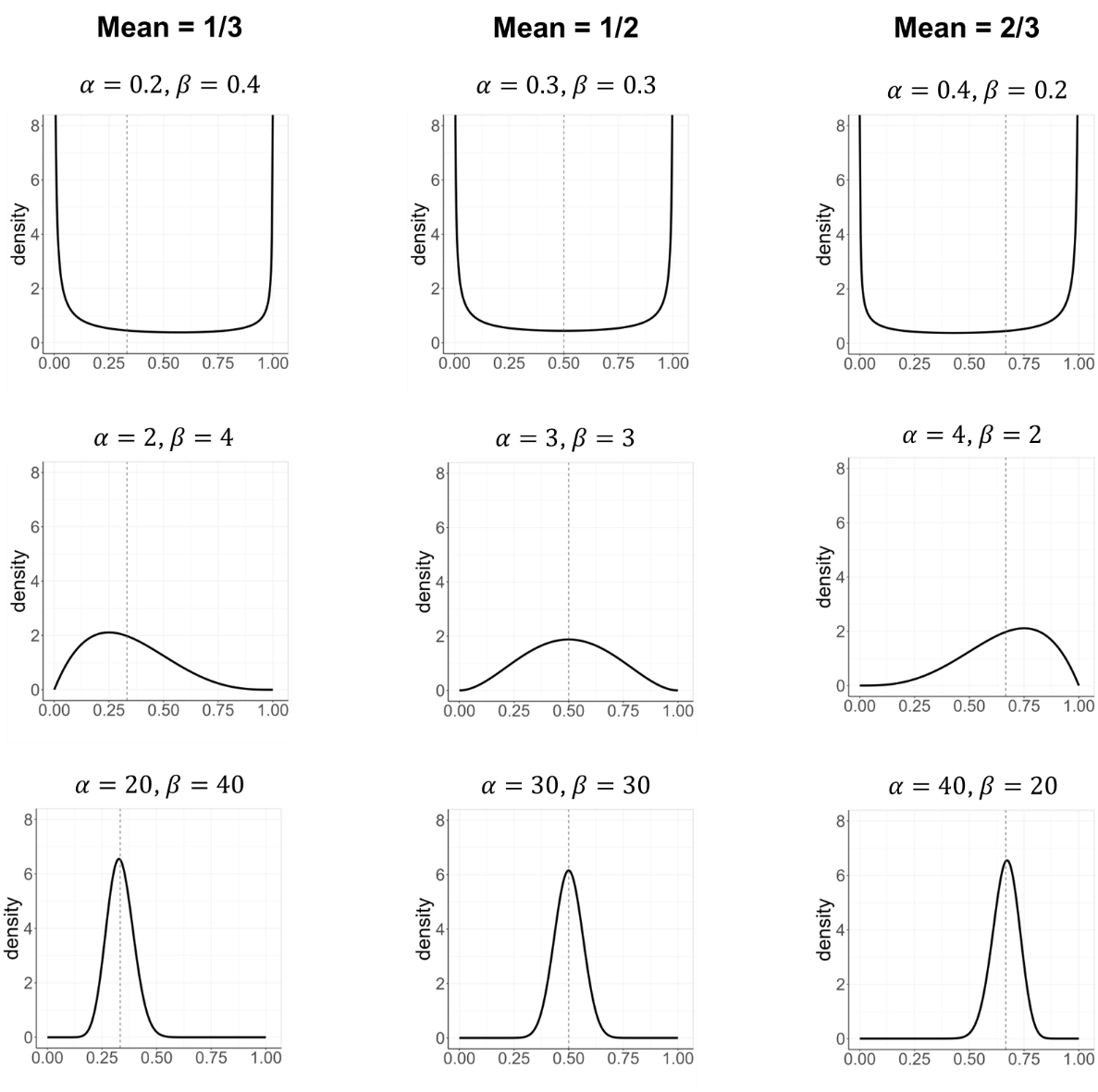


**Fig. S2** Illustration of beta distributions with various parameter values. Gray dashed lines represent the mean of the distribution.

**Model recovery**

To examine if the computational models in our study were identifiable, we generated 50 simulated datasets, each consisting of data of 50 agents, with each model using participants’ trial sequence and random parameter sets derived from uniform distributions. The ranges of the uniform distribution were as follows: $\varepsilon$ (including $\varepsilon_{+}$ and $\varepsilon_{-})$: [0, 1], $\gamma$: [0, 20], $v_{0}$: [0. 1], $\alpha$: [0.08, 32.27], $\beta$: [0.52, 29.67], $\eta$ (including $\eta_{+}$ and $\eta_{-}$): [0, 1], $A$: [0, 1], and $\rho$: [0, 1]. The ranges of $\alpha$ and $\beta$ were determined based on the actual minimum and maximum estimated values of participants. We then conducted model fitting procedures as reported in the main text using the generated datasets and examined the protected exceedance probabilities (PXPs) for each model.

The results are summarized in Fig. S3. All models except for Leaky-$\rho$ model and asymmetrically forgetful Bayesian model showed excellent model recoverability. The Leaky-$\rho$ model was in most cases misidentified as the Leaky model without reward sensitivity parameter, and the asymmetrically forgetful Bayesian model was often misidentified as the forgetful Bayesian model without asymmetry in forgetting effects. However, the basic Bayesian model, which is the best model in the present study, showed high model recoverability both in terms of PXP and model frequency.


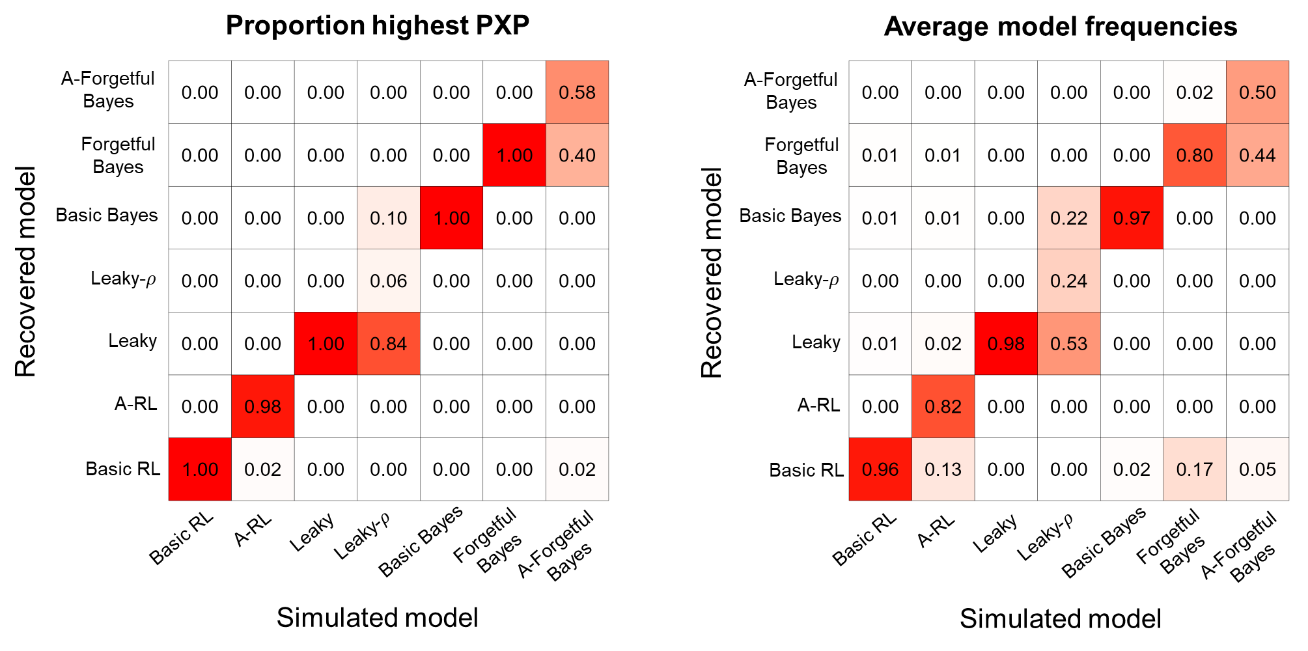


**Fig. S3** Confusion matrices showing the probability of each fitted model given a simulated model. The left panel shows the proportion of the 50 fittings performed on data generated by a simulated model in which given model had the highest protected exceedance probability (PXP). The right panel shows the average model frequencies (note that the model frequency is the proportion of participants best explained by a model) of the 50 fittings performed on data generated by a simulated model. Basic RL: Basic reinforcement learning model, A-RL: Asymmetric reinforcement learning model, Basic Bayes: Basic Bayesian model, Forgetful Bayes: Forgetful Bayesian model, A-Forgetful Bayes: Asymmetrically forgetful Bayesian model.

**Parameter Recovery**

We conducted parameter identifiability analysis with the best model in this study (i.e., Basic Bayesian model). Specifically, first, we pseudo-randomly generated 200 parameter sets using uniform distribution and generated 200 data (each consisting of 300 trials) with each parameter set. The ranges (lower and upper bounds) of uniform distribution were the same as stated in *Model Recovery*. We then conducted model fitting in the same way as stated in the main text using the generated datasets and estimated parameters for each parameter set.

The results of parameter recovery are summarized in Fig. S4. The two parameters of the prior distribution tended to be underestimated compared to the actual values, leading to relatively low correlations between true and estimated values ($\alpha_{0}$: $r$(198) = .45, 95%CI [.33, .55], Fig. S4A; $\beta_{0}$: $r$(198) = .50, 95%CI [.39, .60], Fig. S4B). However, the mean of prior, which is determined by the two parameters and is the main focus of the present study, showed high parameter recoverability ($r$(198) = .92, 95%CI [.89, .94], Fig. S4D). The inverse temperature also showed high recoverability ($r$(198) = .91, 95%CI [.88, .93], Fig. S4C).


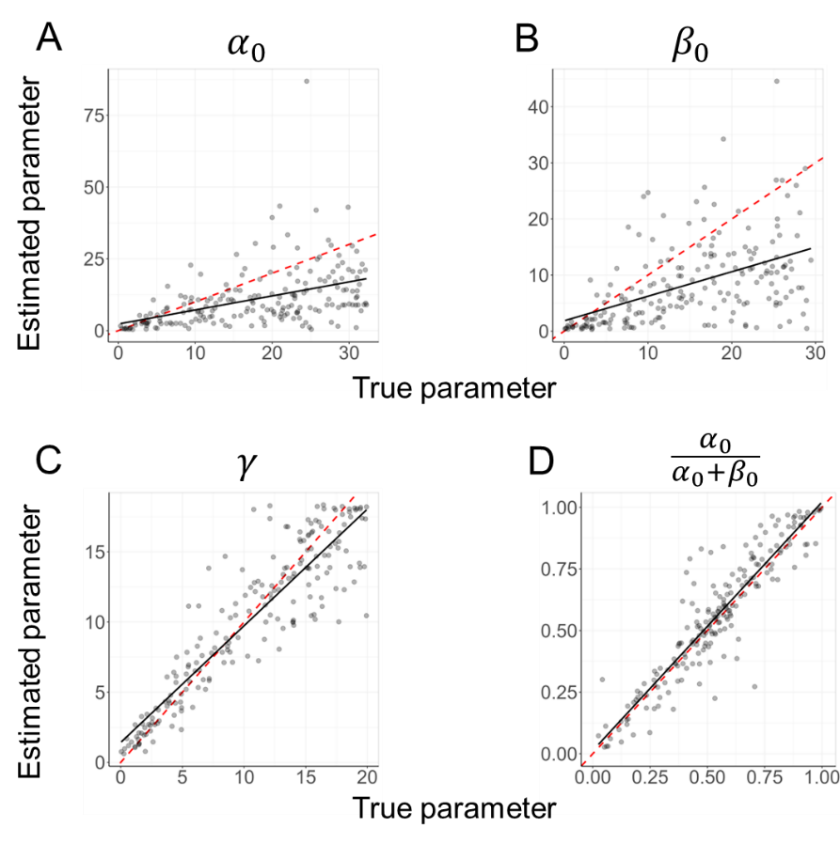


**Fig. S4** Correlation between true (generative) parameters and estimated parameters of Basic Bayesian model. Horizontal axis represents true values of each parameter and vertical axis shows estimated values of each parameter. Red dashed line represents identity line (i.e., y=x), and black line represents best fit linear regression line.

**State optimism at baseline of excluded participants**

As mentioned in the main text, we compared the J-SOM scores at baseline of the excluded participants in two groups, in order to examine the possible bias. The distribution of the score is summarized in Fig. S5 below.


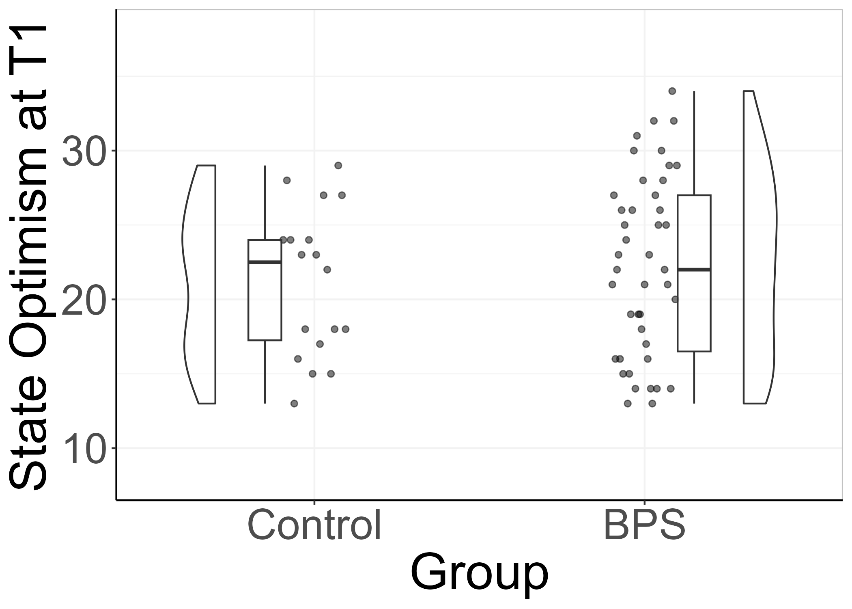


**Fig. S5** The distribution of SOM scores at T1 of excluded participants in control and BPS groups.

**Detailed examination of the belief updating bias**

As mentioned in *Results* and *Discussion*, we obtained little evidence for the positive belief updating bias, unlike previous studies (Kuzmanovic et al., 2017; Sharot et al, 2011). To gain insight into the cause of this result, we conducted several ad-hoc analyses. We first focused on likelihood ratio (LR), which describes how diagnostic the evidence available to a participant is. According to Shah et al. (2016), a LR larger than 1 or smaller than 1, respectively, leads to negatively or positively biased updating in rational Bayesian agents. We first investigated the possibility that the LR of our participants is higher than 1, which may have canceled out the potential true positive updating bias. Based on previous studies, LR was calculated in the following way (Garrett & Sharot, 2017):

$$\begin{aligned} Likelihood Ratio=\frac{\frac{{E1}_{self}}{1-{E1}_{self}}}{\frac{{E1}_{other}}{1-{E1}_{other}}}\#\left( S4 \right) \end{aligned}$$

Note that in this calculation, ${E1}_{self}$ and ${E1}_{other}$ are transformed into decimals ([0, 1]). Participants showed LR larger than 1 in both T1 and T2 (T1: $t\left( 135 \right)=3.20$, $p=.002$, $Mean=1.83$, 95%CI [$1.32$, $2.35$]; T2: $t\left( 135 \right)=3.90$, $p<.001$, $Mean=1.35$, 95%CI [$1.17$, $1.52$]). This could lead to negatively biased update if participants behaved in a similar way to rational Bayesian agents.

To examine the existence of potential true updating bias, we calculated deviance from rational Bayesian updates for each participant (see Box 1 of Garrett & Sharot (2017) for detail). Bayesian posterior odds can be calculated by multiplying prior odds by LR:

$$\begin{aligned} Posterior odds=Prior odds\times LR\#\left( S5 \right) \end{aligned}$$

where prior odds are given by:

$$\begin{aligned} Prior odds= \frac{{E1}_{other}}{1-{E1}_{other}}\#\left( S6 \right) \end{aligned}$$

where ${E1}_{other}$ is expressed as decimals. Using posterior odds, the Bayesian second estimate (in a percentage form) for self can be calculated as follows:

$$\begin{aligned} Bayesian {E2}_{self}=\frac{Posterior Odds}{1+Posterior Odds}\times100\#\left( S7 \right) \end{aligned}$$

Using this value, the Bayesian scaled update can be given by:

$$\begin{aligned} Bayesian {Upd}_{scaled}=\frac{Bayesian {E2}_{self}-{E1}_{self}}{EE}\#\left( S8 \right) \end{aligned}$$

Then, the deviance from Bayesian update was calculated by subtracting the actual participants scaled update from Bayesian scaled update:

$$\begin{aligned} Deviance=Bayesian {Upd}_{scaled}-{Upd}_{scaled}\#\left( S9 \right) \end{aligned}$$

We found that the deviance from Bayesian agents was significantly larger than 0 for both GOOD and BAD conditions in both T1 (GOOD: $t\left( 135 \right)=8.92$, $p<.001$, $Mean=1.93$, 95%CI [$1.50$, $2.36$]; BAD: $t\left( 135 \right)=8.49$, $p<.001$, $Mean=2.17$, 95%CI [$1.66$, $2.68$]; Fig. S5) and T2 (GOOD: $t\left( 135 \right)=8.71$, $p<.001$, $Mean=1.58$, 95%CI [$1.22$, $1.94$]; BAD: $t\left( 135 \right)=8.19$, $p<.001$, $Mean=1.86$, 95%CI [$1.41$, $2.31$]; Fig. S5), meaning that participants updated less than what was expected from the Bayesian model. This result suggests that participants’ behavior cannot be explained by the rational Bayesian model.

Positive belief updating bias in previous studies has been shown to be accounted for by asymmetric deviance from the rational Bayesian model: humans show more deviance when information was BAD compared to GOOD (Garrett & Sharot, 2017; Kuzmanovic et al., 2017). However, paired t-tests showed that the deviance of our participants did not significantly differ depending on whether the news was GOOD or BAD, in both T1 ($t\left( 135 \right)=-0.82$, $p=.41$, $d=-0.09$, $Mean Difference=-0.24$, 95%CI [$-0.81$, $0.34$]; Fig. S5) and T2 ($t\left( 135 \right)=-1.00$, $p=.32$, $d=-0.12$, $Mean Difference=-0.28$, 95%CI [$-0.84$, $0.28$]; Fig. S5).


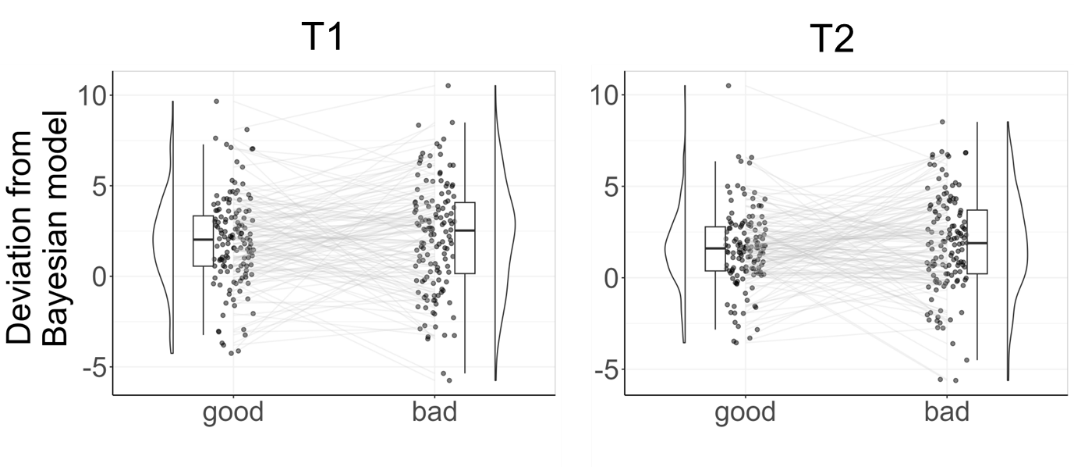


**Fig. S6** Mean deviation of participants’ actual update from that of the rational Bayesian model (left: T1, right: T2). Each point and line correspond to each participant ($n=136$).

**Results regarding belief updating bias in the case where estimation error was calculated by E1_self**

In the main analysis of the news task, we calculated the estimation error (EE) by the difference between the “actual” base rate and the initial estimate for “other” (i.e., $aBR-{E1}_{other}$; Eq. 2), in line with Kuzmanovic et al. (2017). Since individuals cannot directly know the true personal likelihood of experiencing the events, we assumed that participants would update their self-estimates based on information about similar others. However, we can also calculate the difference between aBR and the initial estimate for self (i.e., ${E1}_{self}$). In fact, some of the previous studies have taken this approach (e.g., Sharot et al., 2011; Bottemanne et al., 2022). Therefore, we examined whether the results would differ when we used E1_self rather than E1_other for calculating updating bias.

We found similar results to the original ones (see Fig. S7). Specifically, the BPS intervention did not affect the belief updating bias, indicated by a non-significant interaction between group and time on the bias ($\beta_{group:time}=0.27$, $p=.18$, 95%CI [$-0.12$, $0.67$], Fig. S7A). Also, neither the updating bias at T1 ($\beta=0.07$, $p=.42$, 95%CI [$-0.11$, $0.26$]) nor the change in the updating bias ($\beta=0.12$, $p=.38$, 95%CI [$-0.15$, $0.39$]) showed significant association with the change in state optimism.


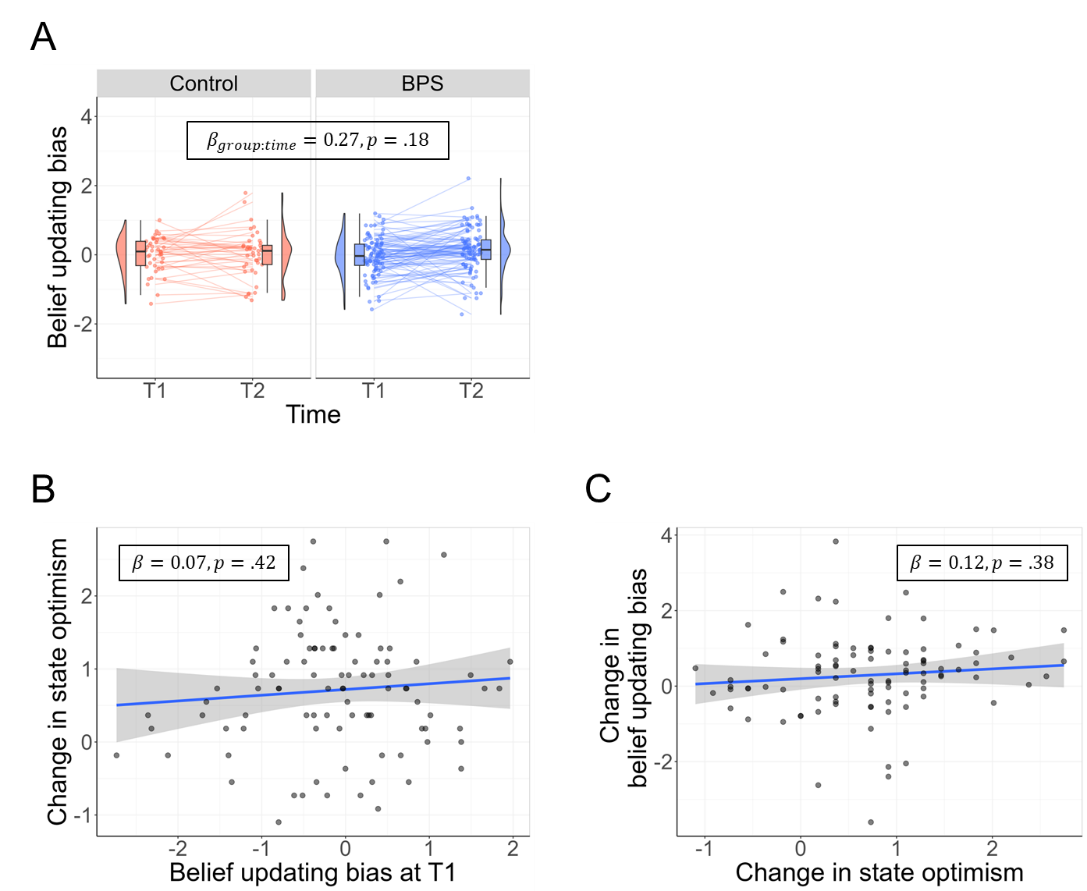


**Fig. S7** Results regarding belied updating bias when E1_self was used instead of E1_other. **A** No effect of the intervention on the updating bias. **B, C** No association between the updating bias at T1 (B) or the change in the updating bias (C) and the change in state optimism.

**Results regarding belief updating bias with less strict exclusion criteria**

In the main analysis of the news task, we excluded participants with more than 50% of trials with no update and more than 20% of trials with odd update (i.e., trials where the sign of estimation error and update do not match; see *2.7.2 Exclusion criteria*). These may have been too harsh, considering the number of total participants excluded with either of these criteria (see Table 2), which may have biased the results. We therefore conducted the same analysis regarding belief updating bias, including participants who were excluded due to the two criteria.

The omission of the two exclusion criteria led to increased sample sizes in both the BPS and control group ($n=114$ for the BPS group and $n=50$ for the control group). We found results consistent with the original ones (see Fig. S8). Specifically, the BPS intervention did not affect the belief updating bias, indicated by a non-significant interaction between group and time on the bias ($\beta_{group:time}=0.23$, $p=.26$, 95%CI [$-0.17$, $0.63$], Fig. S8A). Also, neither the updating bias at T1 ($\beta=0.03$, $p=.73$, 95%CI [$-0.13$, $0.19$]) nor the change in the updating bias ($\beta=-0.06$, $p=.32$, 95%CI [$-0.18$, $0.06$]) showed significant association with the change in state optimism.


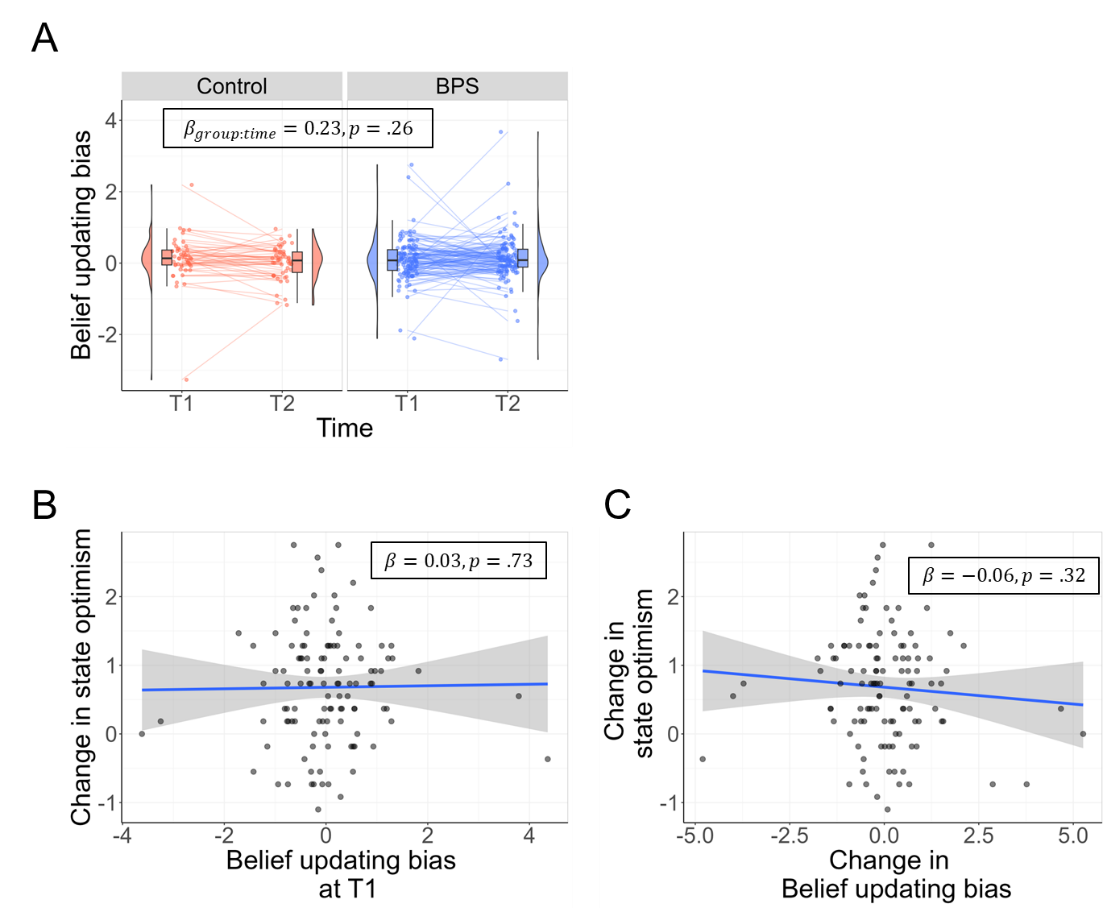


**Fig. S8** Results regarding belied updating bias less strict exclusion criteria. **A** No effect of the intervention on the updating bias. **B, C** No association between the updating bias at T1 (B) or the change in the updating bias (C) and the change in state optimism.

**The association between the change in state optimism and the changes in E1_self and E1_other, in the control group and the overall sample**

As stated in the main text (see 3.3), the change in state optimism showed significant association with the change in E1_self, when analyzed with the control group ($\beta=-0.46$, $p=.04$, 95%CI [$-0.89$, $-0.02$]) and the overall sample ($\beta=-0.27$, $p=.03$, 95%CI [$-0.51$, $-0.03$]). In contrast, it did not show significant association with the change in E1_other, in both the control group ($\beta=-0.37$, $p=.10$, 95%CI [$-0.83$, $0.07$]) and the overall sample ($\beta=-0.13$, $p=.28$, 95%CI [$-0.38$, $0.11$]). These results are visualized in Fig. S9 (see below).


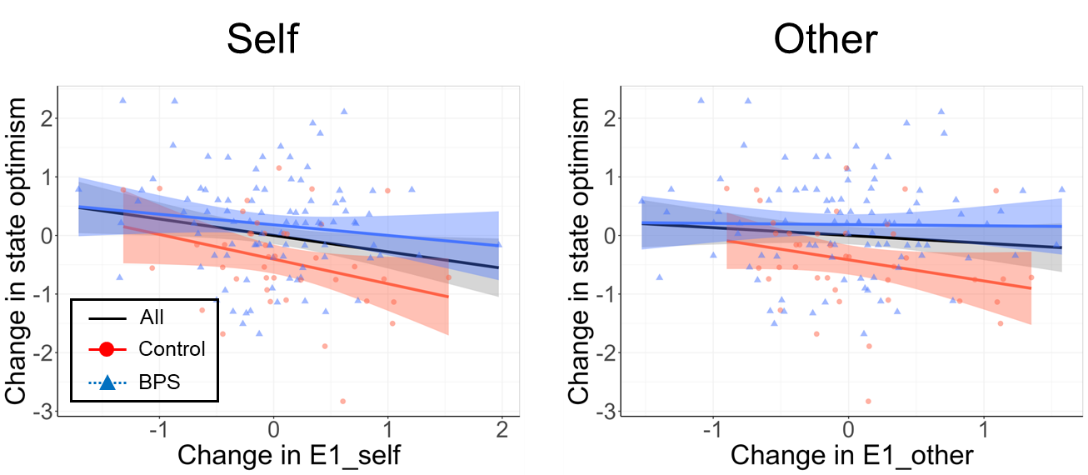


**Fig. S9** The associations between the change in state optimism and the changes in E1_self (left) and E1_other (right). The blue line and points represent the results of the BPS group, the red ones represent the results of the control group, and the black line represents the regression line of the overall sample.

**Results of model comparison in the Pavlovian task**


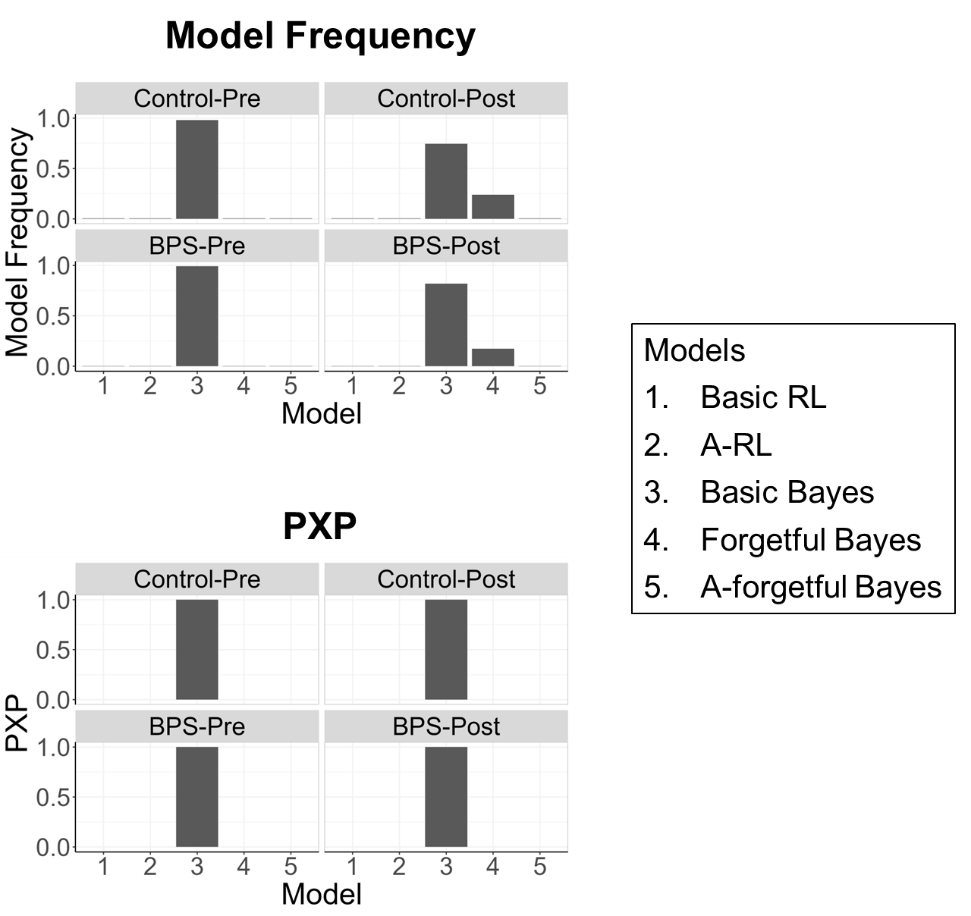


**Fig. S10** Results of model comparison in the Pavlovian task (upper: model frequency, lower: protected exceedance probability). Basic RL: Basic reinforcement learning model, A-RL: Asymmetric reinforcement learning model, Basic Bayes: Basic Bayesian model, Forgetful Bayes: Forgetful Bayesian model, A-Forgetful Bayes: Asymmetrically forgetful Bayesian model. The Basic Bayesian model was estimated to be dominant in all groups and time.

**Results regarding the variance of prior**

As mentioned in the main text, we explored whether the intervention influenced the variance of prior, and whether changes in the variance were associated with changes in state optimism. The variance was quantified using the estimated parameters of the initial Beta distribution in the following way:

$$\begin{aligned} \frac{\alpha_{0}\beta_{0}}{\left( \alpha_{0}+\beta_{0} \right)^{2}\left( \alpha_{0}+\beta_{0}+1 \right)}\#\left( S10 \right) \end{aligned}$$

Results are summarized in the main text. See below (Fig. S6) for visualization of the results and see Table S6 and S12 for detailed results of the regression analyses.


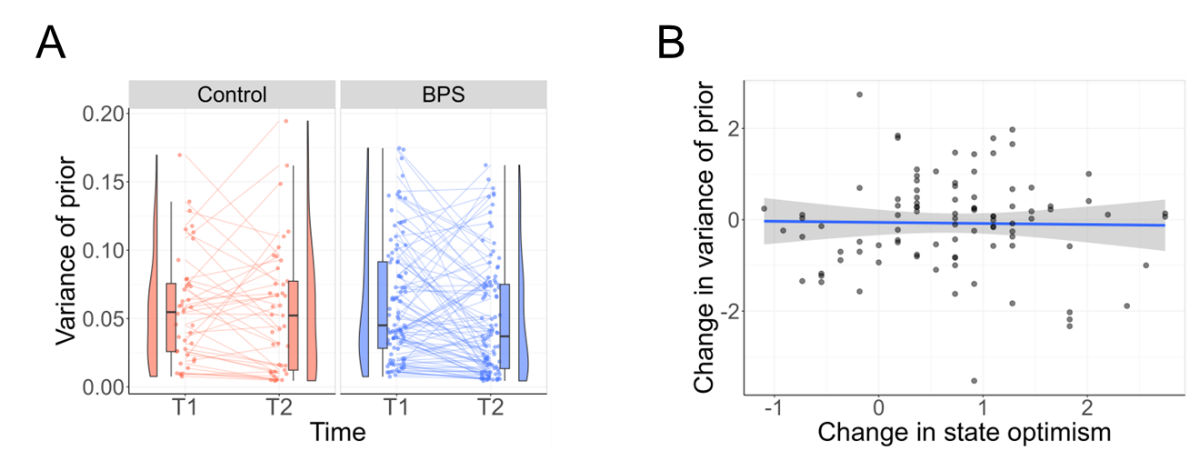


**Fig. S11** Results regarding the variance of prior. A The variance of prior by group and time point. B The association between the change in state optimism and the change in the variance of prior.

**Potential practice effects in the Pavlovian conditioning task**

One of the indices of prior about reward, the shift parameter of sigmoid function, showed an increase in both groups. This suggests that the increase observed in the BPS group may be due to a learning effect from repeatedly encountering the task, rather than the impact of the intervention itself. A similar effect was seen for the inverse temperature, which showed an increasing trend in both groups. Previous research has shown that experiencing the same tasks repeatedly can lead to systematic variations in reinforcement learning parameters (Toyama et al., 2023). In that study, a probabilistic reward learning task was conducted twice with a 1.5-month interval, resulting in increased inverse temperature and perseverance parameters at the second time point (Toyama et al., 2023). Given such systematic changes in behavior, when examining the effects of interventions on behavior using reinforcement learning tasks, it might be better to use a baseline that reflects a state where the task has been encountered before, rather than basing it on the initial experience of the task, to clearly distinguish the effects of interventions from practice effects.

The possible practice effect in the Pavlovian conditioning task casts doubt on the positive association between the intraindividual changes in the mean of prior and state optimism observed in the present study. One could speculate that the positive association observed in the BPS group is just an artifact, which was caused by a coincidental overlap of the increase in state optimism during the intervention and the rise in the mean of prior from practice effects. However, as shown in *Results*, a positive association was selective for the mean of prior. We found no significant association between the change in state optimism and the change in the inverse temperature. If the observed positive association was just a coincidence due to the practice effect, the intraindividual change in state optimism is also likely to correlate with the change in the inverse temperature, which showed an increase at T2 similarly to the shift parameter. In addition, our ad-hoc analysis showed that the positive association between the intraindividual changes in state optimism and the mean of prior remained at the same level when we tested with the data including the control group ($\beta=0.28$, $p=.01$, 95%CI [$0.08$, $0.47$], Fig. S12). Furthermore, when conducting the same analysis using only data from the control group, we obtained the comparable effect size for the association, although the p-value was not small possibly due to a smaller sample size of the control group ($\beta=0.31$, $p=.18$, 95%CI [$-0.14$, $0.76$], Fig. S12). If the positive association seen in the BPS group was due to coincidental, simultaneous increases in state optimism and the prior mean with different causes, the association should be weakened when we included/used the data of the control group, whose population-level state optimism remained the same at T2 as compared to T1. These results thus offer support for the conclusion that there was a positive association between intraindividual changes in state optimism and the prior expectation about reward likelihood.


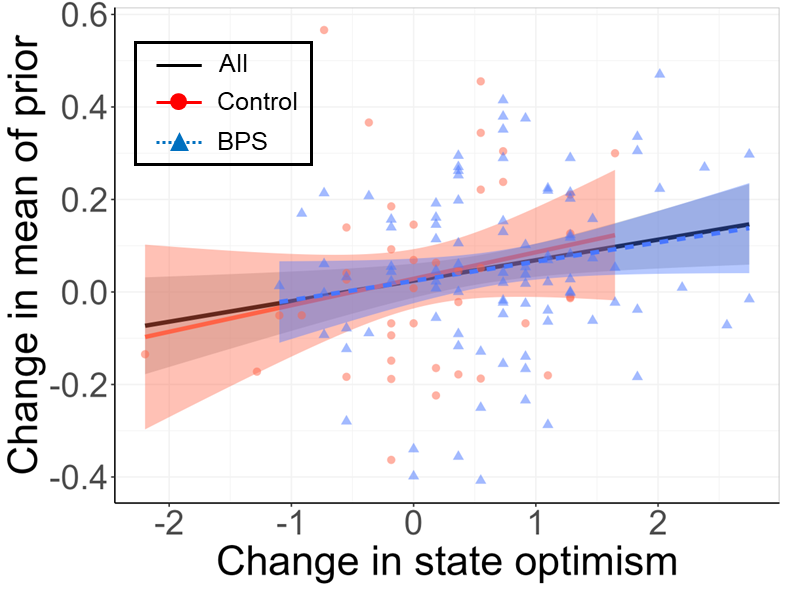


**Fig. S12** The association between the intraindividual changes in state optimism and in mean of prior with data of all participants (black solid line), data of the control group (red circles with a solid line), and the BPS group (blue triangles with a dotted line).

**Supplementary tables**

**Table S1** Number of participants excluded and not excluded in each group

|  | Excluded | Not excluded | Total | %Excluded |
| --- | --- | --- | --- | --- |
| Control | 18 | 41 | 59 | 30.5% |
| BPS | 43 | 95 | 138 | 31.2% |

**Table S2** **Complete list of stimulus events used in the belief updating task**.
We used two lists each consisting of 40 events, and counter-balanced the order of the lists for each participant (i.e., half of participants observed set 1 at T1 and set 2 at T2, while the other half experienced set 2 at T1 and set 1 at T2). The 80 events were collected from the stimuli used in previous studies (Kuzmanovic et al., 2015; Kuzmanovic & Rigoux, 2017; Sharot et al., 2011).

| **List 1** | **List 2** |
| --- | --- |
| fraud when buying something on the internet | death before 60 |
| theft from vehicle | eye cataract (clouding of the lens of the eye) |
| knee osteoarthritis (causing knee pain and swelling) | bicycle theft |
| being cheated by husband/wife | victim of violence by acquaintance |
| more than ￥100,0000 debts | migraine |
| miss a flight | having a stroke |
| death before 80 | victim of violence at home |
| witness a traumatising accident | severe insomnia |
| domestic burglary | osteoporosis (reduced bone density) |
| bone fracture | severe injury due to accident (traffic or house) |
| depression | autoimmune disease |
| heart failure | asthma |
| obesity | blood clot in vein |
| chronic high blood pressure | ulcer |
| diabetes (type 2) | Alzheimer's disease |
| disease of spinal cord | anxiety disorder |
| serious hearing problems | epilepsy |
| infertility | liver disease |
| car stolen | glaucoma |
| dementia | burden by noise |
| gallbladder stones | burden by air pollution |
| appendicitis | burn-out syndrome |
| gluten intolerance | gastric cancer |
| age related blindness | to have a hearing device |
| chronic ringing sound in ear (tinnitus) | increased cholesterol levels |
| alcoholism | working conditions which are detrimental to health |
| Parkinson's disease | urinary incontinence |
| computer crash with loss of important data | heart attack |
| being fired | leukemia |
| skin burn | lung cancer |
| hospital stay longer than three weeks | medical malpractice |
| back pain | being a victim of sexual violence |
| victim of bullying at work (nonphysical) | dependent on nursing care |
| arteries hardening (narrowing of blood vessels) | thyroid disorders |
| theft from person | insufficient pension provision |
| hepatitis A or B | gum problems |
| victim of violence | interruption of a vacation |
| severe teeth problems when old | to be affected by fire |
| colon cancer | being a victim of a sneak thief |
| abnormal heart rhythm | misuse of personal information |

**Table S3 Results of linear mixed regression analysis examining the effect of intervention on state optimism (Eq. 14)**.

| Model: $lmer\left( State Optimism \sim age+sex+group*time+\left( 1 \vert ID \right) \right)$ | | | | | | | |
| --- | --- | --- | --- | --- | --- | --- | --- |
| Fixed effects | | | | | | 95%CI | |
| Name | Beta estimate | SE | t-value | Satterthwaite Approximated DF | p | Lower | Upper |
| Intercept | -0.06 | 0.64 | -0.09 | 136 | .93 | -1.33 | 1.21 |
| Age | 0.02 | 0.03 | 0.08 | 134 | .93 | -0.05 | 0.06 |
| Sex | 0.03 | 0.07 | 0.43 | 150 | .67 | -0.12 | 0.18 |
| Group | -0.38 | 0.18 | -2.09 | 187 | .04 | -0.74 | -0.02 |
| Time | 0.11 | 0.13 | 0.87 | 134 | .38 | -0.14 | 0.36 |
| Group:time | 0.60 | 0.15 | 3.92 | 134 | <.001 | 0.30 | 0.90 |

**Table S4 Results of linear mixed regression analysis examining the effect of intervention on belief updating bias (Eq. 15)**.

| Model: $lmer\left( Upd Bias \sim age+sex+group*time+\left( 1 \vert ID \right) \right)$ | | | | | | | |
| --- | --- | --- | --- | --- | --- | --- | --- |
| Fixed effects | | | | | | 95%CI | |
| Name | Beta estimate | SE | t-value | Satterthwaite Approximated DF | p | Lower | Upper |
| Intercept | 1.07 | 0.60 | 1.79 | 139 | .08 | -0.11 | 2.25 |
| Age | -0.05 | 0.03 | -1.82 | 133 | .07 | -0.10 | 0.00 |
| Sex | -0.06 | 0.07 | -0.86 | 140 | .39 | -0.20 | 0.08 |
| Group | 0.03 | 0.19 | 0.17 | 242 | .87 | -0.34 | 0.40 |
| Time | -0.16 | 0.19 | -0.85 | 134 | .40 | -0.52 | 0.21 |
| Group:time | 0.16 | 0.22 | 0.71 | 134 | .48 | -0.28 | 0.60 |

**Table S5 Results of linear mixed regression analysis examining the effect of intervention on sigmoid shift parameter (Eq. 16)**.

| Model: $lmer\left( Sigmoid Shift \sim age+sex+group*time+\left( 1 \vert ID \right) \right)$ | | | | | | | |
| --- | --- | --- | --- | --- | --- | --- | --- |
| Fixed effects | | | | | | 95%CI | |
| Name | Beta estimate | SE | t-value | Satterthwaite Approximated DF | p | Lower | Upper |
| Intercept | 0.40 | 0.59 | 0.68 | 139 | .50 | -0.76 | 1.56 |
| Age | -0.03 | 0.03 | -1.08 | 133 | .28 | -0.08 | 0.02 |
| Sex | -0.21 | 0.07 | -3.10 | 140 | .002 | -0.35 | -0.08 |
| Group | 0.11 | 0.18 | 0.60 | 238 | .55 | -0.25 | 0.47 |
| Time | 0.40 | 0.18 | 2.26 | 134 | .03 | 0.05 | 0.74 |
| Group:time | -0.07 | 0.21 | -0.32 | 134 | .75 | -0.48 | 0.35 |

**Table S6 Results of linear mixed regression analysis examining the effect of intervention on the mean of prior belief on reward likelihood (Eq. 17)**.

| Model: $lmer\left( Prior Mean \sim age+sex+group*time+\left( 1 \vert ID \right) \right)$ | | | | | | | |
| --- | --- | --- | --- | --- | --- | --- | --- |
| Fixed effects | | | | | | 95%CI | |
| Name | Beta estimate | SE | t-value | Satterthwaite Approximated DF | p | Lower | Upper |
| Intercept | 0.49 | 0.57 | 0.86 | 139 | .39 | -0.64 | 1.63 |
| Age | -0.03 | 0.02 | -1.04 | 132 | .30 | -0.07 | 0.02 |
| Sex | -0.25 | 0.07 | -3.71 | 132 | <.001 | -0.39 | -0.12 |
| Group | -0.02 | 0.18 | -0.12 | 243 | .90 | -0.38 | 0.34 |
| Time | 0.21 | 0.18 | 1.19 | 134 | .24 | -0.14 | 0.57 |
| Group:time | 0.11 | 0.22 | 0.53 | 134 | .60 | -0.31 | 0.54 |

**Table S7 Results of linear mixed regression analysis examining the effect of intervention on the variance of prior (Eq. 22)**.

| Model: $lmer\left( Prior Variance \sim age+sex+group*time+\left( 1 \vert ID \right) \right)$ | | | | | | | |
| --- | --- | --- | --- | --- | --- | --- | --- |
| Fixed effects | | | | | | 95%CI | |
| Name | Beta estimate | SE | t-value | Satterthwaite Approximated DF | p | Lower | Upper |
| Intercept | 0.47 | 0.64 | 0.73 | 136 | .47 | -0.81 | 1.74 |
| Age | -0.02 | 0.03 | -0.71 | 132 | .48 | -0.07 | 0.04 |
| Sex | 0.03 | 0.08 | 0.45 | 132 | .65 | -0.12 | 0.19 |
| Group | 0.11 | 0.19 | 0.56 | 206 | .56 | -0.26 | 0.48 |
| Time | -0.04 | 0.15 | -0.28 | 134 | .78 | -0.34 | 0.26 |
| Group:time | -0.24 | 0.18 | -1.32 | 134 | .19 | -0.60 | 0.12 |

**Table S8 Results of linear mixed regression analysis examining the effect of intervention on the inverse temperature (Eq. 23)**.

| Model: $lmer\left( Inverse Temperature \sim age+sex+group*time+\left( 1 \vert ID \right) \right)$ | | | | | | | |
| --- | --- | --- | --- | --- | --- | --- | --- |
| Fixed effects | | | | | | 95%CI | |
| Name | Beta estimate | SE | t-value | Satterthwaite Approximated DF | p | Lower | Upper |
| Intercept | -1.54 | 0.60 | -2.58 | 138 | .01 | -2.72 | -0.36 |
| Age | 0.06 | 0.03 | 2.23 | 132 | .03 | 0.01 | 0.11 |
| Sex | 0.02 | 0.07 | 0.25 | 132 | .80 | -0.12 | 0.16 |
| Group | 0.14 | 0.19 | 0.74 | 234 | .46 | -0.05 | 0.64 |
| Time | 0.30 | 0.18 | 1.70 | 134 | .09 | -0.23 | 0.50 |
| Group:time | -0.00 | 0.21 | -0.02 | 134 | .99 | -0.42 | 0.41 |

**Table S9 Results of multiple regression analysis examining the association between intraindividual changes in state optimism and belief updating bias at T1 (Eq. 18)**.

| Model: $lm\left( {State Optimism}_{T1\to T2} \sim age+sex+{Upd Bias}_{T1} \right)$ | | | | | | | |
| --- | --- | --- | --- | --- | --- | --- | --- |
| Name | Beta estimate | SE | t-value | DF | p | 95%CI  Lower | 95%CI  Upper |
| Intercept | -0.00 | 0.75 | -0.00 | 91 | 1.00 | -1.49 | 1.49 |
| Age | 0.03 | 0.03 | 0.98 | 91 | .33 | -0.03 | 0.10 |
| Sex | -0.14 | 0.09 | -1.58 | 91 | .12 | -0.31 | 0.03 |
| Upd Bias_T1_ | -0.05 | 0.10 | -0.51 | 91 | .61 | -0.25 | 0.15 |

**Table S10 Results of multiple regression analysis examining the association between intraindividual changes in state optimism and belief updating bias (Eq. 19)**.

| Model: $lm\left( {Upd Bias}_{T1\to T2} \sim age+sex+{State Optimism}_{T1\to T2} \right)$ | | | | | | | |
| --- | --- | --- | --- | --- | --- | --- | --- |
| Name | Beta estimate | SE | t-value | DF | p | 95%CI  Lower | 95%CI  Upper |
| Intercept | -2.83 | 1.08 | -2.62 | 91 | .01 | -4.97 | -0.68 |
| Age | 0.13 | 0.05 | 2.74 | 91 | .01 | 0.04 | 0.22 |
| Sex | 0.09 | 0.13 | 0.68 | 91 | .50 | -0.17 | 0.34 |
| SO_T1→T2_ | -0.18 | 0.16 | -1.18 | 91 | .24 | -0.49 | 0.13 |

*SO: state optimism

**Table S11 Results of multiple regression analysis examining the association between intraindividual changes in state optimism and sigmoid Shift Parameter (Eq. 20)**.

| Model: $lm\left( {Sigmoid Shift}_{T1\to T2} \sim age+sex+{State Optimism}_{T1\to T2} \right)$ | | | | | | | |
| --- | --- | --- | --- | --- | --- | --- | --- |
| Name | Beta estimate | SE | t-value | DF | p | 95%CI  Lower | 95%CI  Upper |
| Intercept | 2.80 | 0.95 | 2.96 | 91 | .004 | 0.92 | 4.68 |
| Age | -0.12 | 0.04 | -2.88 | 91 | .005 | -0.20 | -0.04 |
| Sex | 0.13 | 0.11 | 1.12 | 91 | .27 | -0.10 | 0.35 |
| SO_T1→T2_ | 0.35 | 0.14 | 2.56 | 91 | .01 | 0.08 | 0.62 |

*SO: state optimism

**Table S12 Results of multiple regression analysis examining the association between intraindividual changes in state optimism and the mean of prior belief on reward likelihood (Eq. 21)**.

| Model: $lm\left( {Prior Mean}_{T1\to T2} \sim age+sex+{State Optimism}_{T1\to T2} \right)$ | | | | | | | |
| --- | --- | --- | --- | --- | --- | --- | --- |
| Name | Beta estimate | SE | t-value | DF | p | 95%CI  Lower | 95%CI  Upper |
| Intercept | 2.25 | 0.80 | 2.80 | 91 | .006 | 0.65 | 3.85 |
| Age | -0.11 | 0.04 | -3.03 | 91 | .003 | -0.18 | -0.04 |
| Sex | 0.09 | 0.10 | 0.92 | 91 | .37 | -0.10 | 0.28 |
| SO_T1→T2_ | 0.29 | 0.12 | 2.45 | 91 | .02 | 0.06 | 0.52 |

*SO: state optimism

**Table S13 Results of multiple regression analysis examining the association between intraindividual changes in state optimism and the inverse temperature (Eq. 28)**.

| Model: $lm\left( {Prior Variance}_{T1\to T2} \sim age+sex+{State Optimism}_{T1\to T2} \right)$ | | | | | | | |
| --- | --- | --- | --- | --- | --- | --- | --- |
| Name | Beta estimate | SE | t-value | DF | p | 95%CI  Lower | 95%CI  Upper |
| Intercept | 0.28 | 0.91 | 0.30 | 91 | .76 | -1.53 | 2.08 |
| Age | -0.01 | 0.04 | -0.42 | 91 | .68 | -0.10 | 0.06 |
| Sex | 0.17 | 0.11 | 1.53 | 91 | .13 | -0.05 | 0.38 |
| SO_T1→T2_ | 0.02 | 0.13 | 0.14 | 91 | .89 | -0.24 | 0.28 |

*SO: state optimism

**Table S14 Results of multiple regression analysis examining the association between intraindividual changes in state optimism and the inverse temperature (Eq. 29)**.

| Model: $lm\left( {Inverse Temperature}_{T1\to T2} \sim age+sex+{State Optimism}_{T1\to T2} \right)$ | | | | | | | |
| --- | --- | --- | --- | --- | --- | --- | --- |
| Name | Beta estimate | SE | t-value | DF | p | 95%CI  Lower | 95%CI  Upper |
| Intercept | -0.72 | 0.93 | -0.77 | 91 | .44 | -2.56 | 1.12 |
| Age | 0.03 | 0.04 | 0.76 | 91 | .45 | -0.05 | 0.11 |
| Sex | -0.18 | 0.11 | -1.61 | 91 | .11 | -0.40 | 0.04 |
| SO_T1→T2_ | -0.04 | 0.13 | 0.29 | 91 | .77 | -0.23 | 0.31 |

*SO: state optimism

**Table S15 Results of linear mixed regression analysis examining the effect of intervention on E1_self (Eq. 22)**.

| Model: $lmer\left( E1_{self} \sim age+sex+group*time+\left( 1 \vert ID \right) \right)$ | | | | | | | |
| --- | --- | --- | --- | --- | --- | --- | --- |
| Fixed effects | | | | | | 95%CI | |
| Name | Beta estimate | SE | t-value | Satterthwaite Approximated DF | p | Lower | Upper |
| Intercept | -0.35 | 0.69 | -0.51 | 138 | .61 | -1.72 | 1.02 |
| Age | 0.02 | 0.03 | 0.75 | 136 | .46 | -0.04 | 0.08 |
| Sex | 0.02 | 0.08 | 0.28 | 170 | .78 | -0.13 | 0.18 |
| Group | 0.001 | 0.19 | 0.004 | 160 | .99 | -0.37 | 0.37 |
| Time | -0.26 | 0.10 | -2.65 | 134 | .01 | -0.45 | -0.06 |
| Group:time | -0.09 | 0.12 | -0.82 | 134 | .42 | -0.32 | 0.13 |

**Table S16 Results of linear mixed regression analysis examining the effect of intervention on E1_other (Eq. 23)**.

| Model: $lmer\left( E1_{other} \sim age+sex+group*time+\left( 1 \vert ID \right) \right)$ | | | | | | | |
| --- | --- | --- | --- | --- | --- | --- | --- |
| Fixed effects | | | | | | 95%CI | |
| Name | Beta estimate | SE | t-value | Satterthwaite Approximated DF | p | Lower | Upper |
| Intercept | -0.64 | 0.69 | -0.93 | 137 | .35 | -2.01 | 0.72 |
| Age | 0.03 | 0.03 | 1.13 | 136 | .26 | -0.03 | 0.09 |
| Sex | 0.04 | 0.08 | 0.49 | 168 | .62 | -0.12 | 0.19 |
| Group | 0.01 | 0.19 | 0.06 | 161 | .96 | -0.36 | 0.38 |
| Time | -0.23 | 0.10 | -2.33 | 134 | .02 | -0.42 | -0.03 |
| Group:time | -0.06 | 0.12 | -0.52 | 134 | .60 | -0.29 | 0.17 |

**Table S17 Results of multiple regression analysis examining the association between intraindividual changes in state optimism and E1_self (Eq. 24)**.

| Model: $lm\left( {E1_{self}}_{T1\to T2} \sim age+sex+{State Optimism}_{T1\to T2} \right)$ | | | | | | | |
| --- | --- | --- | --- | --- | --- | --- | --- |
| Name | Beta estimate | SE | t-value | DF | p | 95%CI  Lower | 95%CI  Upper |
| Intercept | -0.62 | 0.75 | -0.84 | 91 | .41 | -2.11 | 0.86 |
| Age | 0.04 | 0.03 | 1.11 | 91 | .27 | -0.02 | 0.10 |
| Sex | -0.12 | 0.09 | -1.41 | 91 | .16 | -0.30 | 0.05 |
| SO_T1→T2_ | -0.15 | 0.14 | -1.07 | 91 | .29 | -0.43 | 0.13 |

*SO: state optimism

**Table S18 Results of multiple regression analysis examining the association between intraindividual changes in state optimism and E1_other (Eq. 25)**.

| Model: $lm\left( {E1_{other}}_{T1\to T2} \sim age+sex+{State Optimism}_{T1\to T2} \right)$ | | | | | | | |
| --- | --- | --- | --- | --- | --- | --- | --- |
| Name | Beta estimate | SE | t-value | DF | p | 95%CI  Lower | 95%CI  Upper |
| Intercept | -0.66 | 0.75 | -0.87 | 91 | .39 | -2.15 | 0.84 |
| Age | 0.04 | 0.03 | 1.15 | 91 | .26 | -0.03 | 0.10 |
| Sex | -0.13 | 0.09 | -1.51 | 91 | .14 | -0.31 | 0.04 |
| SO_T1→T2_ | -0.01 | 0.14 | -0.05 | 91 | .96 | -0.28 | 0.27 |

*SO: state optimism
